# How Reproducible are Electrochemical Impedance Spectroscopic Data for Dye-Sensitized Solar Cells?

**DOI:** 10.3390/ma13071547

**Published:** 2020-03-27

**Authors:** Mariia Becker, Maria-Sophie Bertrams, Edwin C. Constable, Catherine E. Housecroft

**Affiliations:** Department of Chemistry, University of Basel, BPR 1096, Mattenstrasse 24a, CH-4058 Basel, Switzerland; mariia.karpacheva@unibas.ch (M.B.); maria-sophie.bertrams@stud.unibas.ch (M.-S.B.); edwin.constable@unibas.ch (E.C.C.)

**Keywords:** electrochemical impedance spectroscopy, dye-sensitized solar cells, dye, statistical analysis

## Abstract

Dye-sensitized solar cell (DSC) technology has been broadly investigated over the past few decades. The sandwich-type structure of the DSC makes the manufacturing undemanding under laboratory conditions but results in the need for reproducible measurements for acceptable DSC characterization. Electrochemical impedance spectroscopy (EIS) offers the possibility to study complex electronic systems and is commonly used for solar cells. There is a tendency in the literature to present impedance data only for one representative device. At the same time, as current density–voltage plots illustrate, measurements can vary within one set of DSCs with identical components. We present multiple DSC impedance measurements on “identical” devices prepared using two different dyes and present a statistical analysis regarding the reproducibility.

## 1. Introduction

Over the last few decades, solar cells have become a key technology due to their ability to convert sunlight into electrical energy. Silicon-based solar cells are already established in the marketplace, but their costly and tedious fabrication motivates the examination of alternative systems. Dye-sensitized solar cells (DSCs) are a promising solution. The sandwich-type DSC structure is easy to manufacture with a broad variety of sensitizers and offers lower-cost devices. The photoanode typically consists of a fluorine-doped tin oxide (FTO)-coated glass substrate with a layer of a sintered nanoparticulate semiconductor (usually TiO_2_ in n-type DSCs), onto which a sensitizer is adsorbed.

The high scientific interest in DSCs has resulted in the development of techniques for measuring their performance. The necessity for optimized and reproducible measurements is critical. The most broadly used characterization methods for DSCs are current density–voltage plots (*J*–*V* curves), external quantum efficiency (EQE) measurements, and electrochemical impedance spectroscopy (EIS). The solar-to-electrical energy conversion efficiency (*η*) is a solar-irradiance-dependent unit, which can be written as Equation (1) [1]:

(1) $$\eta =\frac{P_{\mathrm{OUT}}}{P_{\mathrm{IN}}}=\frac{\left(J\mathrm{sc}\right)\left(V\mathrm{oc}\right)\left(ff\right)}{P_{\mathrm{IN}}},$$

where *η* is presented as a function of the short-circuit current density (*J*_SC_), the open-circuit voltage (*V*_OC_), the fill factor (*ff*), and total incident solar power of the cell (*P*_IN_). Due to the relationship between *η* and *P*_IN_, the correct characterization of DSCs depends not only on the calibration of the irradiance to a standard 1 Sun at an air mass (AM) of 1.5, but also on the active area of the DSC, which needs to be smaller than the total surface of the area of TiO_2_ [2,3]. This is achieved by masking the cell, such that only a small circular region of a functionalized semiconductor is irradiated. Use of unmasked cells without a set irradiance area results in an overestimation of *η*. 

Incident photon-to-current conversion efficiency (IPCE) or EQE is the spectral response of a solar cell to the light in terms of current [4]. The IPCE can be calculated according to Equation (2) [4]:

(2) $$IPCE=\frac{rate\;number\;of\;collected\;electrons}{rate\;number\;of\;collected\;photons}.$$

For accurate measurements, factors including the calibrated light at 1 Sun, cell masking, and temperature control for long measurements have to be considered. 

**Figure 1:**
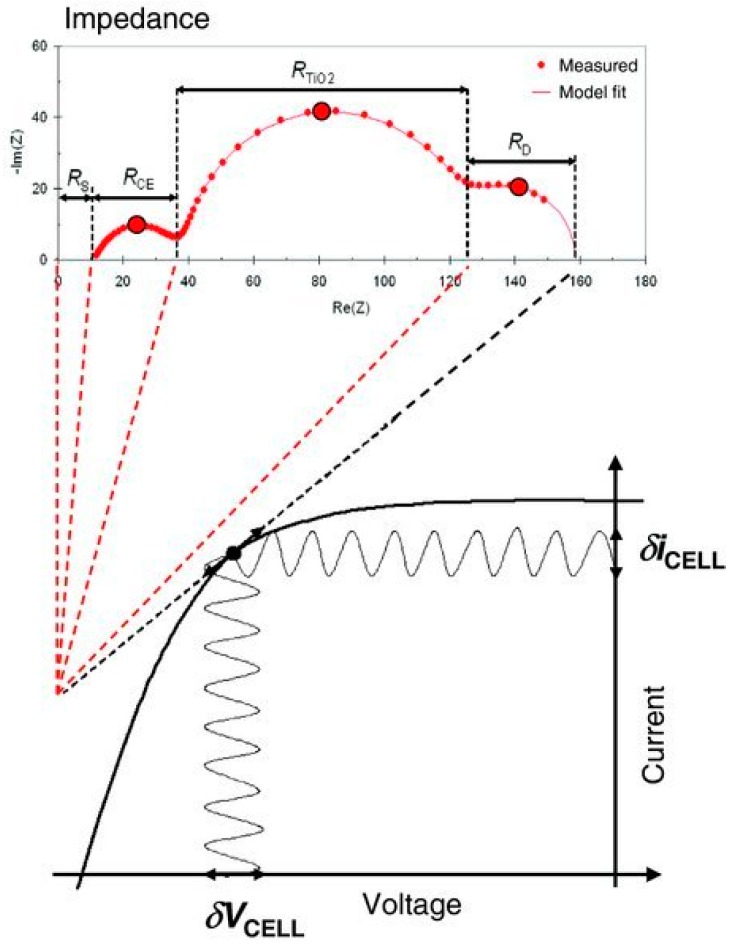
Schematic representation of the relationship between a current–voltage (*I*–*V*) plot and its differential resistances measured by electrochemical impedance spectroscopy (EIS). Individual differential resistance components of the *I*–*V* curve appear as separate impedance arcs due to their different characteristic frequencies. Reproduced with permission from Reference [8].

Electrochemical impedance spectroscopy is a well-known technique used for studying electrical properties of different materials [5,6]. A DSC has a complicated structure involving multiple interfaces, at which many electronic processes take place simultaneously. In contrast to resistance, impedance is not limited to one circuit element and can therefore be used to describe a system as a more complex and general circuit. EIS is based on the application of a small alternating current (AC) perturbation (*Ṽ*) at a fixed frequency (*f*) over a sample in equilibrium at stationary bias [7]. The impedance at a specific frequency is denoted as *Z*(f) and is presented in Equation (3):

(3) $$Z\left(f\right)=\frac{Ṽ}{Ĩ},$$

where *Ĩ* is an AC modulation. The impedance can be seen as a frequency-dependent differential resistance of the *I*–*V* curve, because of the small amplitude of the AC voltage modulation (Figure 1) [8]. For the full impedance spectrum, a measurement is made in a frequency range from hertz (Hz) to kilohertz (kHz). 

**Figure 2:**
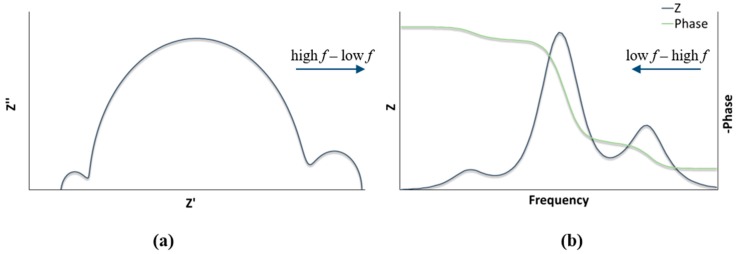
(**a**) Nyquist plot representation; (**b**) Bode plot representation.

EIS results are typically presented in Nyquist and Bode plots, of which the fitting results in parameters including the series resistance (R_s_), the resistance (R_Pt_), and the capacitance (C_Pt_) of a counter electrode, the recombination resistance (R_rec_), the chemical capacitances (C_μ_), and the diffusion resistances of charge carriers in an electrolyte (Ws). Impedance plots include the real (Z’) and imaginary (Z’’) parts. Classically, a Nyquist plot consists of three semicircles at an open circuit potential (Figure 2a). The high-frequency region before the beginning of the curve depicts the series resistance. The first semicircle at high *f* is associated with the counter electrode, the second semicircle is associated with the semiconductor–electrolyte interface, and the last semicircle at low *f* is associated with the diffusion of the electrolyte. The resistance value can be estimated by the width of the arc along the abscissa [9]. The Bode plot provides an important representation of resistances from the plateaus (Figure 2b). The electron lifetime (τ) is a critical parameter, which is inversely proportional to the maximum frequency (*f*_max_), and can be extracted from the Bode plot [10].

EIS spectra can be recorded with different irradiation intensities, circuit conditions, and frequency ranges. Under carefully chosen conditions, a significant number of essential processes can be distinguished according to the spectral shapes of an impedance response, including electron transport in TiO_2_, electron recombination at a TiO_2_–electrolyte interface, and charge transfer at a counter electrode [11]. Data extracted from *J*–*V* curves can be explained in more detail with the help of EIS. For example, EIS parameters including the recombination resistance, chemical capacitance, transport resistance, and diffusion length contribute to the value of *J*_SC_ for a DSC. This allows for a more precise explanation about the limiting factors of the DSC performance. Many studies have been reported in order to investigate the correct interpretation of EIS results [12,13,14,15,16,17]. 

**Figure 3:**
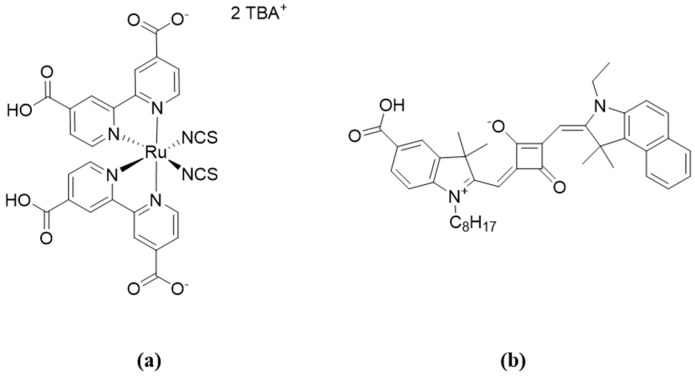
(**a**) Structure of N719 (TBA^+^ = tetra-*n*-butylammonium); (**b**) structure of SQ2.

On the other hand, to the best of our knowledge, there is no discussion in the literature of how reproducible EIS results for DSCs are. Usually, published data refer only to one measured cell. It is a common, but by no means universal, practice for current density–voltage measurements to be presented in the literature for two or more cells. A reproducibility study of DSCs with the standard dye N719 showed only a small deviation in *η* of 5.76 ± 0.14% [18]. In order to broaden this investigation to gain insight into the reproducibility of EIS measurements for DSCs, we performed the impedance analysis of DSCs functionalized with two commercially available dyes, N719 and SQ2. The Nyquist profiles are considerably different for N719 and SQ2 (Figure 3) [19,20,21], and in this paper, we examine the impedance reproducibility for DSCs sensitized with a metal complex and an organic dye. 

## 2. Materials and Methods 

DSC fabrication is described in the Supporting Information. The dipping time of the working electrodes for N719 in a dye bath containing the dye was 16 h, and that for SQ2 was 1 h [18]. The electrolyte contained an I_3_^−^/I^−^ redox shuttle and had a composition of 0.1 M LiI, 0.05 M I_2_, 0.5 M 1-methylimidazole, and 0.6 M 1-butyl-3-methylimidazolium iodide in 3-methoxypropionitrile for both N719- and SQ2-based DSCs.

## 3. Results and Discussion

### 3.1. Effect of the Preirradiation of DSCs

**Figure 4:**
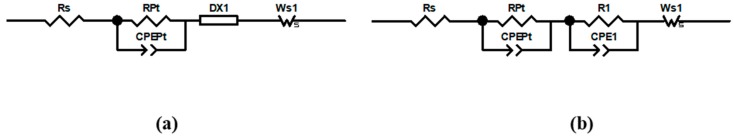
The two different equivalent circuit models used in this study: (**a**) the equivalent circuit model 1 used for fitting N719-sensitized dye-sensitized solar cells (DSCs); (**b**) the equivalent circuit model 2 used for fitting the DSCs with N719 or SQ2 as a sensitizer.

In order to study the reproducibility of the EIS experiments, we measured two sets of DSCs with dyes N719 and SQ2. For the fitting of the N719 set, we used the equivalent circuit model 1 (Figure 4a). This circuit model 1 consisted of five elements and included a series resistance (Rs), a resistance (R_Pt_), and a constant phase element (CPE_Pt_) to model a counter electrode, an extended distributed element (DX1) to represent a mesoporous TiO_2_/electrolyte interface as a transmission line model, and a Warburg element (Ws), which represented the diffusion of the electrolyte. The transmission line model is broadly used for the fitting of DSCs impedance plots. Unfortunately, it is not always suitable. In the case of the SQ2 set, a simplified equivalent circuit model was needed (model 2), which did not have a DX1 element (Figure 4b). Instead, elements R1 and CPE1 modeled a recombination charge transfer resistance and a chemical capacitance. The usage of model 1 will result in extremely high transport resistance values, which will be greater than recombination resistance. This effect showed that transmission line model could not be used. In our study, a constant phase element is employed because of the roughness of the surface [5,22]. For a realistic comparison between the N719- and SQ2-based DSCs, we also used model 2 for fitting the DSCs with the N719 dye. Thus, we can compare the reproducibility of sets with two different dyes and the reproducibility of EIS experiments in terms of two models—circuit model 1 and circuit model 2 in the case of the N719-based DSCs.

**Figure 5:**
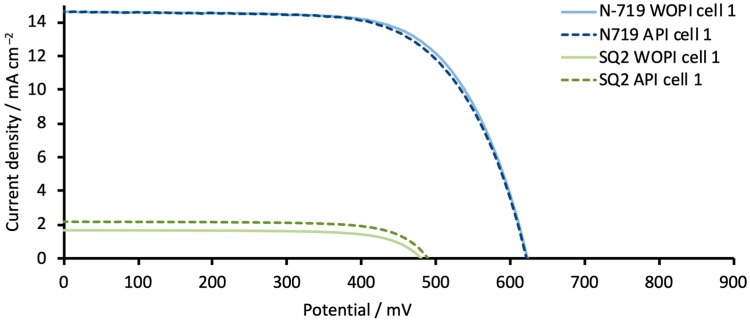
*J*–*V* curves for dyes N719 (blue) and SQ2 (green) measured WOPI and after a preirradiation time (API) of 15 min. The data for duplicate DSCs and the *J*–*V* parameters are presented in Figure S1 and Table S1 (Supplementary Materials).

The chemical capacitance was calculated according to Equation (4):

(4) $$C_{\mu }={\left(R_{\mathrm{rec}}{}^{1-\alpha }Q\right)}^{1/\alpha }$$

where *Q* is a prefactor of CPE and *α* is an empirical constant. Our first goal was to compare and understand the reproducibility of the DSCs during EIS measurements. It is known that the *J*–*V* performance and CPE are dependent on the preirradiation of DSCs before measurements [23]. In 2013, Nguyen et al. published a work where they performed light stress EIS measurements within a period of 6 to 175 h [24]. It was shown that the impedance spectra significantly changed depending on the time under irradiation. Thus, we decided to test if the preirradiation of cells is also beneficial for EIS. We measured two DSCs with N719 and two DSCs with SQ2. The experiments were performed directly after the fabrication of DSCs without preirradiation, (WOPI), and each DSC was measured 5 times with a break of 15 min between measurements. Then, in the same manner, we measured the same DSC after a preirradiation time (API) of 15 min with a light intensity of 1 Sun at 1.5 AM (100 mW cm^−2^). The *J*–*V* parameters with and without preirradiation are shown in Figure 5, Figure S1, and Table S1 (Supplementary Materials). Between the EIS experiments, the DSCs were kept under ambient light. 

According to the data in Table 1, the time when the EIS measurements were made after building the DSCs has an impact on the impedance measurements. We observed that the DSCs with SQ2 were more affected by the waiting time before the measurements than the N719-based cells. For the N719-based cells, R_rec_ was only slightly affected by standing for four periods of 15 min (hereafter, referred to as 60 min) after cell fabrication, and the values changed only from 26 to 22 Ω. On the other hand, the values of C_μ_ changed from 845 to 970 μF. τ values were only slightly variable and were in a range of 22 to 18 ms. Ws and parameters for the counter electrode stayed constant. In the case of the SQ2-based DSCs, we could clearly observe a trend that, after each 15 min period allowing the DSCs to stand in ambient light, R_rec_ significantly decreased. The R_rec_ values reduced from 185 to 94 Ω, which were reflected in the values of C_μ_ and Ws. At the same time, the counter electrode was affected with R_Pt_ changing from 32 to 19 Ω, when the standing time increased from 0 to 60 min after the DSC fabrication.

For both DSC sets, the main difference between 0 and 60 min under ambient light after cell building lied in the recombination resistance and capacitance values. For both sensitizers, the recombination resistance decreased with the standing time. The value of C_μ_ depended on the values of R_rec_, *Q*, and *α* according to Equation (4). The values of *α* stayed constant over the 60 min period, and the values of *Q* had only a small variation (Table S2, Supplementary Materials). Thus, R_rec_ had the highest impact on C_μ_, and its lower values resulted in a higher chemical capacitance. 

It is known [12] that R_rec_ values are lower under irradiation than in the dark. Our next step was to measure DSCs after preirradiation. According to the data in Figure 4 and Table S1 (Supplementary Materials), the *J*–*V* curves for the N719-based DSCs were not affected by the preirradiation, in contrast to those for the SQ2-based cells. For the N719-based DSCs, R_rec_ changed from 19 to 22 Ω from 0 to 60 min API (Table 2). These resistance values were very similar to the values obtained between 15 and 60 min after DSC fabrication WOPI. The C_μ_ values between 0 and 60 min API have only a small variation (976 to 990 μF) compared to those WOPI. For the SQ2 dye, we observed the opposite trend. The increase in *J*_SC_ results in a higher overall performance, which is reflected in the impedance of the cells. For 0 min and 60 min API, C_μ_ values change little (37 to 42 μF), while the difference in R_rec_ values is greater than 10 Ω. Other parameters including τ, Ws and counter electrode parameters, stay constant. 

### 3.2. General Reproducibility of DSCs in Terms of EIS Measurements

**Figure 6:**
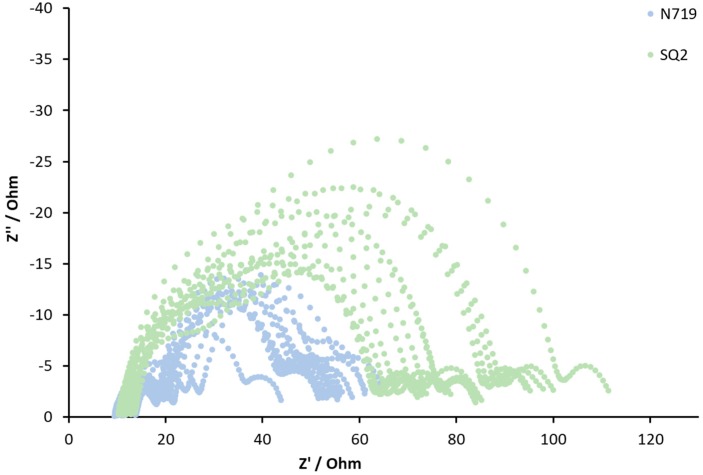
Nyquist plots of the DSCs with N719 (blue) and SQ2 (green) sensitizers. The plots represent the experimental data.

To check the impedance reproducibility of DSCs and to confirm how many cells should be measured in order to obtain an overview of representative cell impedance for a target system, we measured 15 DSCs containing N719 and 15 cells with SQ2 for APIs in a range of 45–60 min. The experimental data are presented in Figure 6, and the fitted data are shown in Figures S2–S4 (Supplementary Materials). For the N719-containing DSCs, three semicircles in each Nyquist plot could be clearly separated from each other. In the case of the SQ2-containing DSCs, the first two semicircles overlapped, which made the electron transport resistance indistinguishable from R_rec_. Thus, we used model 2 (Figure 4b) for fitting the experimental data. 

As mentioned above, in the case of the N719-containing DSCs, we fitted the EIS curves using both models 1 and 2. Since model 1 is commonly used, we showed that the data in Table S3 (Supplementary Materials) and Table 3 revealed that the fitting with model 1 compared to that using model 2 did not result in significant differences in values of the recombination resistance (R_rec_), chemical capacitance (C_μ_), empirical constant (*α*), electron lifetime (τ), and Warburg resistance (Ws). The platinum counter electrode fittings were also comparable for both models. Thus, our further statistical analysis is suitable for model 1 as well. Data for *J*–*V* measurements are presented in Table S4 and Figure S5 (Supplementary Materials).

In Table 4, statistical data for the N719-based DSCs are presented. These include the maximum (max) and minimum (min) values, average value, SD, and relative standard deviation (RSD). Statistical data were extracted from the fitted EIS parameters. The average value was a sum of all the values for a given parameter divided by 15. SD is a statistical value, which gives information about the dispersion of a data set values. RSD is a ratio of the SD to the average and expressed as a percentage (Equations (S1)–(S3), Supplementary Materials).

**Figure 7:**
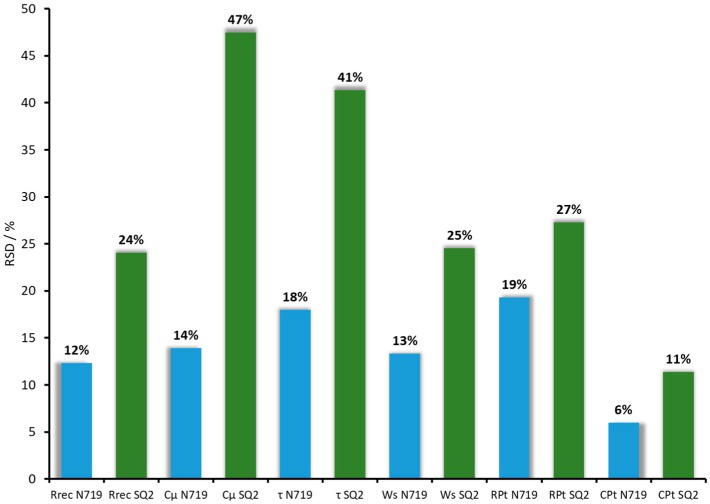
The overview of RSDs for N719 and SQ2 dyes. The blue bars refer to N719, and the green bars represent SQ2.

The analysis of the N719-based DSCs allowed us to assess the statistical difference between 15 cells. The RSD values for R_rec_, C_μ_, τ, Ws, R_Pt_, and C_Pt_ were in a range of 14% to 19% (Figure 7). The max value in the case of R_rec_ was 30 Ω, while the min value was 17 Ω, giving an average value of 24 Ω. Within the set of DSCs with N719, only three cells differed from the average value by more than 3 Ω. This showed that the RSD value of 12% was mainly due to a few outlying cells, and most of the DSCs had good reproducibility in terms of recombination resistance. The values of C_μ_ were not as reproducible as R_rec_ and ranged from 604 to 971 μF. Thus, the SD of the chemical capacitance was ±113 μF, and the average of the chemical capacitance values was 816 μF, giving an RSD of 14%. The average value of Ws was 11 Ω, and the SD of Ws was ±1 Ω. Most of the cells had Ws values between 9 and 12 Ω with one DSC having a Ws value of 14 Ω. This resulted in an RSD value of 13%. The highest RSDs were observed for τ and R_Pt_ with 18% and 19%, respectively. Since the values of τ depended on R_rec_ and C_μ_ [25], the range of τ values was rather broad (14 to 26 ms). The counter electrode values are usually considered as constant and should have minimal deviation from cell to cell. Despite this, the DSCs in this investigation exhibited an RSD of R_Pt_ of 19%. Most of the cells had R_Pt_ values in a range of 9 ± 2 Ω, but two cells had R_Pt_ values of 13 Ω. The values of C_Pt_ had the smallest RSD of 6% and lied between 5 and 6 μF. Nonetheless, the fact that there were outlying cells underlined the importance of acquiring EIS data for a series of cells and not for a single device. In order to understand whether the SD values for N719 could be considered as rather high or low, we performed the same study for the SQ2 dye (Figures S6–S8, Supplementary Materials).

Compared to the DSCs containing N719, the SQ2-based DSCs exhibited a lower reproducibility in terms of overall performance because of the significant differences in values of *J*_SC_ (Table S5 and Figure S9, Supplementary Materials). The same trend was observed in the EIS measurements. In Table 5, the EIS parameters for 15 DSCs with SQ2 as a sensitizer are presented. 

The statistical analysis of the EIS parameters confirmed the lower reproducibility for the SQ2-contaning DSCs compared to those of the N719-containing devices (Table 6 and Figure 6). The R_rec_ values ranged between 72 and 33 Ω with an average value of 45 Ω. The SD and RSD values for the SQ2-based DSCs were ±11 Ω and 24%, respectively, while those for the N719-based DSCs were 3 Ω and 12%, respectively. The C_μ_ values varied widely (93 to 23 μF) with an average value of 47 μF, leading to an RSD of 47%. Since R_rec_ and C_μ_ are related to *J*_SC_, their high RSD values could explain the low reproducibility of *J*_SC_, which varied from 3.94 to 2.14 mA cm^−2^. The maximum and minimum values of electron lifetime were 3 and 1 ms, respectively (τ_average_ = 2 ms). This led to an SD of ±1 ms and an RSD of 41%. The Ws had only one outlying cell with a value of 2 Ω, which led to an RSD value of 25% for the set of 15 DSCs. The rest of the DSCs had Ws values within a range of 8 to 11 Ω. 

The R_Pt_ values of the counter electrodes varied more significantly (12 to 30 Ω) for the SQ2-based DSCs than for the N719-based DSCs. The SD was ±5 Ω, and the RSD was 27%. At the same time, C_Pt_ stayed more or less constant and had an SD of ±1 μF with an average value of 5 μF, resulting in an RSD of 11%.

**Figure 8:**
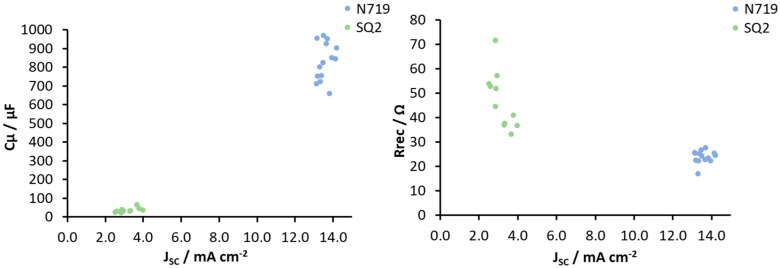
C_μ_ and R_rec_ presented as a function of *J*_SC_ for the DSCs with the N719 and SQ2 dyes.

C_μ_ and R_rec_ represented the shifts of the conduction band and the electron injection rate, which affected the *J*_SC_ values. In Figure 8, the values of C_μ_ and R_rec_ are presented as a function of *J*_SC_ for both the N719 and SQ2 dyes, since all three parameters were related to one other. The data for the two dyes form separate groups on the graph and can be easily distinguished from each other, despite the rather high RSD values of C_μ_ and R_rec_ for the SQ2-containing DSCs. 

## 4. Conclusions

Our findings lead to the conclusion that it is important to perform EIS measurements on multiple cells to ensure representative data are collected about the electronic processes in DSCs. SQ2 is a good example of a dye which has a wide variability and illustrates how diverse the parameters can be within one set of devices with identical components and fabricated in the same manner. There is a strong tendency in the DSC literature to present EIS data with only one device for a given dye, and the conclusions based on these data may be erroneous. Thus, we encourage the DSC research community to perform EIS measurements on at least four cells in order to determine reasonable average values. 

## Figures and Tables

**Table 1:** The EIS parameters for the DSCs with N719 and SQ2 dyes. The DSCs were measured without preirradiation (WOPI).

DSC ^1^	R_rec_ (Ω)	C_μ_ (μF)	τ (ms)	Ws (Ω)	R_s_ (Ω)	R_Pt_ (Ω)	C_Pt_ (μF)
N719 WOPI 0 min	26	845	22	11	15	7	4
N719 WOPI 15 min	22	905	20	11	13	8	4
N719 WOPI 30 min	19	955	18	10	13	8	4
N719 WOPI 45 min	22	981	22	10	12	8	5
N719 WOPI 60 min	22	970	21	10	12	8	5
SQ2 WOPI 15 min	185	18	3	15	12	32	9
SQ2 WOPI 30 min	136	20	3	11	12	23	6
SQ2 WOPI 45 min	107	22	2	11	11	20	6
SQ2 WOPI 60 min	94	23	2	10	11	19	5

^1^ The DSC SQ2 WOPI 0 min was not fitted due to high resistance values, which resulted in a high error of the fitting.

**Table 2:** The EIS parameters for the DSCs with N719 and SQ2 dyes. The DSCs were measured with 15 min preirradiation under 1 sun at a 1.5 air mass (AM) (100 mW cm^−2^). Between the measurements, DSCs were kept under ambient light.

DSC	R_rec_ (Ω)	C_μ_ (μF)	τ (ms)	Ws (Ω)	R_s_ (Ω)	R_Pt_ (Ω)	C_Pt_ (μF)
N719 API 0 min	19	976	19	11	11	9	5
N719 API 15 min	23	948	22	10	11	9	5
N719 API 30 min	20	991	20	10	11	9	5
N719 API 45 min	20	1007	20	10	11	9	5
N719 API 60 min	22	990	21	10	13	9	5
SQ2 API 0 min	65	37	2	10	10	17	5
SQ2 API 15 min	62	37	2	9	10	18	5
SQ2 API 30 min	56	39	2	9	10	17	5
SQ2 API 45 min	48	42	2	9	10	16	5
SQ2 API 60 min	52	42	2	9	10	17	5

**Table 3:** The EIS parameters for DSCs with N719 dye at an API range (45–60 min), which were fitted with model 2. The Nyquist and Bode plots are presented in Figures S2–S4 (Supplementary Materials).

DSC	R_rec_ (Ω)	C_μ_ (μF)	*α*	τ (ms)	Ws (Ω)	R_s_ (Ω)	R_Pt_ (Ω)	C_Pt_ (μF)
N719 cell 1	25	756	0.92	19	12	12	13	4
N719 cell 2	22	724	0.95	16	11	11	9	4
N719 cell 3	24	902	0.94	22	9	14	8	4
N719 cell 4	22	852	0.94	19	10	11	9	4
N719 cell 5	26	847	0.92	22	11	10	9	4
N719 cell 6	24	971	0.94	24	9	11	7	5
N719 cell 7	25	955	0.94	24	14	14	8	4
N719 cell 8	23	927	0.93	21	11	10	10	4
N719 cell 9	28	951	0.94	26	11	9	8	5
N719 cell 10	27	826	0.93	22	11	14	13	4
N719 cell 11	22	753	0.94	17	10	11	9	4
N719 cell 12	17	801	0.95	14	9	12	6	4
N719 cell 13	24	660	0.94	16	10	10	9	4
N719 cell 14	26	711	0.93	18	10	10	10	4
N719 cell 15	30	604	0.93	18	9	11	9	4

**Table 4:** Statistical data for the N719-based DSCs extracted from the fitted EIS parameters.

Parameter	R_rec_ (Ω)	C_μ_ (μF)	τ (ms)	Ws (Ω)	R_Pt_ (Ω)	C_Pt_ (μF)
Maximum (max)	30	971	26	14	13	5
Minimum (min)	17	604	14	9	6	4
Average	24	816	20	11	9	4
SD	3	113	4	1	2	0.3
Relative standard deviation (RSD) (%)	12	14	18	13	19	6

**Table 5:** The EIS parameters for the DSCs with SQ2 dye in an API range (45–60 min), which were fitted with model 2. The Nyquist and Bode plots are presented in Figures S6–S8 (Supplementary Materials).

DSC	R_rec_ (Ω)	C_μ_ (μF)	*α*	τ (ms)	Ws (Ω)	R_s_ (Ω)	R_Pt_ (Ω)	C_Pt_ (μF)
SQ2 cell 1	45	39	0.81	2	8	11	20	4
SQ2 cell 2	53	30	0.80	2	2	12	12	5
SQ2 cell 3	54	26	0.79	1	8	10	12	5
SQ2 cell 4	37	32	0.82	1	11	11	19	5
SQ2 cell 5	52	27	0.81	1	11	11	26	5
SQ2 cell 6	37	36	0.82	1	9	10	16	4
SQ2 cell 7	72	23	0.80	2	11	13	17	5
SQ2 cell 8	38	35	0.82	1	10	11	14	5
SQ2 cell 9	33	66	0.83	2	9	11	21	4
SQ2 cell 10	57	35	0.81	2	10	10	20	5
SQ2 cell 11	41	45	0.82	2	11	12	19	5
SQ2 cell 12	34	93	0.86	3	9	13	24	4
SQ2 cell 13	45	59	0.88	3	10	11	30	4
SQ2 cell 14	44	77	0.89	3	11	14	27	4
SQ2 cell 15	34	78	0.85	3	10	12	17	4

**Table 6:** Statistical data for the SQ2-based DSCs extracted from the fitted EIS parameters.

Parameter	R_rec_ (Ω)	C_μ_ (μF)	τ (ms)	Ws (Ω)	R_Pt_ (Ω)	C_Pt_ (μF)
max	72	93	3	11	30	5
min	33	23	1	2	12	4
average	45	47	2	9	20	5
SD	11	22	1	2	5	1
RSD (%)	24	47	41	25	27	11

## Supplementary Materials

The following are available online at https://www.mdpi.com/1996-1944/13/7/1547/s1, Table S1: *J*–*V* parameters for the DSCs with the N719 and SQ2 dyes WOPI and API; Table S2: Experimental parameters α and Q needed for the correction of the constant phase element (CPE) and the final capacitance for the DSCs with the N719 and SQ2 dyes WOPI and API; Table S3: The EIS parameters for the DSCs with the N719 dye with fitting model 1; Table S4: *J*–*V* parameters for the DSCs with the N719 dye; Table S5: *J*–*V* parameters for the DSCs with the SQ2 dye; Figure S1: *J*–*V* curves for the DSCs with the dyes N719 and SQ2 WOPI and API; Figure S2: EIS data for the DSCs with the N719 dye (cells 1–5); Figure S3: EIS data for the DSCs with the N719 dye (cells 6–10); Figure S4: EIS data for the DSCs with the N719 dye (cells 11–15); Figure S5: *J*–*V* curves for the DSCs with the N719 dye; Figure S6: EIS data for DSCs with SQ2 dye (cells 1–5); Figure S7: EIS data for the DSCs with the SQ2 dye (cells 6–10); Figure S8: EIS data for the DSCs with the SQ2 dye (cells 11–15); Figure S9: *J*–*V* curves for the DSCs with the SQ2 dye; Equation (S1): Average value; Equation (S2): SD value; Equation (S3): RSD value; Equations (1)–(3) are used to calculate average, SD, and RSD values, respectively.

## References

[1] Bozic-Weber B., Constable E.C., Housecroft C.E. (2013). Light Harvesting with Earth Abundant d-Block Metals: Development of Sensitizers in Dye-Sensitized Solar Cells (DSCs). Coord. Chem. Rev..

[2] Snaith H.J. (2012). The perils of solar cell efficiency measurements. Nat. Photon..

[3] Snaith H.J. (2012). How should you measure your excitonic solar cells?. Energy Environ. Sci..

[4] Pazoki M., Cappel U.B., Johansson E.M.J., Hagfeldt A., Boschloo G. (2017). Characterization techniques for dye-sensitized solar cells. Energy Environ. Sci..

[5] Liberatore M., Decker F., Burtone L., Zardetto V., Brown T.M., Reale A., Di Carlo A. (2009). Using EIS for diagnosis of dye-sensitized solar cells performance. J. Appl. Electrochem..

[6] Fabregat-Santiago F., Bisquert J., Palomares E., Otero L., Kuang D., Zakeeruddin S.M., Grätzel M. (2007). Correlation between Photovoltaic Performance and Impedance Spectroscopy of Dye-Sensitized Solar Cells Based on Ionic Liquids. J. Phys. Chem. C.

[7] Tian H., Boschloo G., Hagfeldt A. (2018). Molecular Devices for Solar Energy Conversion and Storage.

[8] Halme J., Vahermaa P., Miettunen K., Lund P. (2010). Device physics of dye solar cells. Adv. Mater..

[9] Bhatt P., Pandey K., Yadav P., Tripathi B., Kumar M. (2016). Impedance Spectroscopic Investigation of the Degraded Dye-Sensitized Solar Cell due to Ageing. Int. J. Photoenergy.

[10] Ho P., Bao L.Q., Ahn K.-S., Cheruku R., Kim J.H. (2016). P-Type dye-sensitized solar cells: Enhanced performance with a NiO compact blocking layer. Synth. Met..

[11] Wang Q., Ito S., Grätzel M., Fabregat-Santiago F., Mora-Seró I., Bisquert J., Bessho T., Imai H. (2006). Characteristics of High Efficiency Dye-Sensitized Solar Cells. J. Phys. Chem. B.

[12] Fabregat-Santiago F., Bisquert J., Garcia-Belmonte G., Boschloo G., Hagfeldt A. (2005). Influence of electrolyte in transport and recombination in dye-sensitized solar cells studied by impedance spectroscopy. Sol. Energy Mater. Sol. Cells.

[13] Sacco A. (2017). Electrochemical impedance spectroscopy: Fundamentals and application in dye-sensitized solar cells. Renew. Sustain. Energy Rev..

[14] Wei-Qing L., Zhong-Guan L., Dong-Xing K., Lin-Hua H., Song-Yuan D. (2013). Wide frequency range diagnostic impedance behavior of the multiple interfaces charge transport and transfer processes in dye-sensitized solar cells. Electrochim. Acta.

[15] Bisquert J. (2011). A variable series resistance mechanism to explain the negative capacitance observed in impedance spectroscopy measurements of nanostructured solar cells. Phys. Chem. Chem. Phys..

[16] Fabregat-Santiago F., Garcia-Belmonte G., Mora-Seró I., Bisquert J. (2011). Characterization of nanostructured hybrid and organic solar cells by impedance spectroscopy. Phys. Chem. Chem. Phys..

[17] Bisquert J. (2003). Chemical capacitance of nanostructured semiconductors: Its origin and significance for nanocomposite solar cells. Phys. Chem. Chem. Phys..

[18] Klein Y.M., Willgert M., Perscimone A., Constable E.C., Housecroft C.E. (2016). Positional isomerism makes a difference: Phosphonic acid anchoring ligands with thienyl spacers in copper(I)-based dye-sensitized solar cells. Dalton Trans..

[19] Sarker S., Seo H.W., Kim D.M. (2013). Electrochemical impedance spectroscopy of dye-sensitized solar cellswith thermally degraded N719 loaded TiO_2_. Chem. Phys. Lett..

[20] Wei L., Yang Y., Fan R., Wang P., Li L., Yu J., Yang B., Cao W. (2013). Enhance the performance of dye-sensitized solar cells by co-sensitization of 2,6-bis(iminoalkyl)pyridine and N719. RSC Adv..

[21] Malzner F.J., Willgert M., Constable E.C., Housecroft C.E. (2017). The way to panchromatic copper(I)-based dyesensitized solar cells: Co-sensitization with the organic dye SQ2. J. Mater. Chem. A..

[22] Han L., Koide N., Chiba Y., Islam A., Mitate T. (2006). Modeling of an equivalent circuit for dye-sensitized solar cells: Improvement of efficiency of dye-sensitized solar cells by reducing internal resistance. Comptes Rendus Chim..

[23] Yeoh M.E., Chan K.Y. (2019). Short-term light soaking effect on dye-sensitized solar cells. J. Phys. Conf. Ser..

[24] Nguyen H.T., Tran H.M., Nguyen T.T.P. (2013). Application of Electrochemical Impedance Spectroscopy in Characterization of Mass- and Charge Transfer Processes in Dye-Sensitized Solar Cells. ECS Trans..

[25] Bisquert J., Fabregat-Santiago F., Mora-Seró I., Garcia-Belmonte G., Giménez S. (2009). Electron Lifetime in Dye-Sensitized Solar Cells: Theory and Interpretation of Measurements. J. Phys. Chem. C.

