# Supplementary material for: How Reproducible are Electrochemical Impedance Spectroscopic Data for Dye-Sensitized Solar Cells?
Source: Materials (Basel). 2020 Mar 27;13(7):1547. doi: 10.3390/ma13071547 (PMC7178167; doi:10.3390/ma13071547)
Supplement: Supplementary file 1 [file materials-13-01547-s001.pdf]

Supporting information

for

How reproducible are electrochemical impedance spectroscopic data for dye-sensitized solar cells?

Mariia Becker, Maria-Sophie Bertrams, Edwin C. Constable and Catherine E. Housecroft

Department of Chemistry, University of Basel, BPR 1096, Mattenstrasse 24a, CH-4058 Basel, Switzerland; mariia.karpacheva@unibas.ch (M.B.); maria-sophie.bertrams@stud.unibas.ch (M.-S.B.); edwin.constable@unibas.ch (E.C.C.); catherine.housecroft@unibas.ch (C.E.H.)

Contents

DSC fabrication.. 3

DSC measurements.. 3

Table S1. *J-V* parameters for DSCs with N719 and SQ2 dyes WOPI and API. 4

Figure S1. *J-V* curves for dyes N719 and SQ2 WOPI and API. 4

Table S2. Experimental parameters *α* and Q needed for the correction of CPE and the final capacitance for DSCs with N719 and SQ2 dyes WOPI and API. 4

Table S3. The EIS parameters for DSCs with N719 dye with fitting model 1. 5

Figure S2. EIS data for DSCs with N719 dye (cells 1-5). 5

Figure S3. EIS data for DSCs with N719 dye (cells 6-10). 6

Figure S4. EIS data for DSCs with N719 dye (cells 11-15). 7

Table S4. *J-V* parameters for DSCs with N719 dye. 8

Figure S5. *J-V* curves for DSCs with N719 dye.. 8

Equation S1. Average value. 9

Equation S2. Standard deviation value.. 9

Equation S3. Relative standard deviation value. 9

Figure S6. EIS data for DSCs with SQ2 dye (cells 1-5). 10

Figure S7. EIS data for DSCs with SQ2 dye (cells 6-10). 11

Figure S8. EIS data for DSCs with SQ2 dye (cells 11-15). 12

Table S5. *J-V* parameters for DSCs with SQ2 dye. 13

Figure S9. *J-V* curves for DSCs with SQ2 dye.. 13

DSC fabrication

Each working commercial TiO_2 electrode (opaque, Solaronix) was rinsed with EtOH and dried on a heating plate at 450 °C for 30 min. The electrodes were cooled to 60 °C and dipped in an 0.3 mM EtOH solution of N719 (Solaronix) overnight. In case of SQ2 dye electrodes were immersed in an 0.1 mM CH_2Cl_2 solution of the dye for 1 h. After soaking in the dye-baths, the electrodes were washed with the same solvent as used in the dye-bath and dried with a heat gun.

Commercial platinum counter electrodes from Solaronix (Test Cell Platinum Electrodes Drilled) were rinsed with EtOH and dried on a heating plate at 450 °C for 30 min. The TiO_2 electrodes and Pt counter-electrodes were assembled together using thermoplast hot-melt sealing foil (Solaronix, Test Cell Gaskets, made from Meltonix 1170-60 sealing film, 60 microns thick) by heating them together. The vacuum backfilling technique was used to introduce the electrolyte into DSCs through a hole drilled in the counter electrode and this was then sealed with a cover glass using hot-melt sealing foil.

DSC measurements

The solar cell measurements used fully masked cells using black coloured copper sheet with a single aperture placed over the screen printed dye-sensitized TiO_2 square. The area of the aperture in the mask was smaller than the active area of the dye-sensitized TiO_2 (0.36 cm^2). For complete masking, black cover was also applied over the edges and rear of the cell. Current density-voltage (*J*–*V*) measurements were made by irradiating from the photoanode side with a LOT Quantum Design LS0811 instrument (100 mW cm^{-2} = 1 sun at AM 1.5) and the simulated light power was calibrated with a silicon reference cell.

The EQE measurements were performed on a Spe-Quest quantum efficiency setup from ReRa Systems (Netherlands) equipped with a 100W halogen lamp (QTH) and a lambda 300 grating monochromator from Lot Oriel. The monochromatic light was modulated to 1 Hz using a chopper wheel from ThorLabs. The cell response was amplified with a large dynamic range IV converter from CVI Melles Griot and then measured with a SR830 DSP Lock-In amplifier from Stanford Research.

For the EIS measurements a ModuLab® XM PhotoEchem photoelectrochemical measurement system from Solartron Analytical was used. The impedance was measured at the open-circuit potential of the cell at a light intensity of 22 mW cm^{-2} (590 nm) in the frequency range 0.05 Hz to 100 kHz using an amplitude of 10 mV. The impedance data were analysed and fitted using ZView® sofware from Scribner Associates Inc.

Table S1. *J-V* parameters for DSCs with N719 and SQ2 dyes WOPI and API.

DSC	J_{SC} / mA cm^{-2}	V_{OC} / mV	*ff* / %	η / %
N719 WOPI cell 1	14.64	622	68	6.17
N719 WOPI cell 2	12.84	603	69	5.33
N719 API cell 1	14.62	621	66	6.04
N719 API cell 2	12.70	613	67	5.24
SQ2 WOPI cell 1	1.07	465	70	0.35
SQ2 WOPI cell 2	1.67	480	71	0.57
SQ2 API cell 1	1.74	477	71	0.58
SQ2 API cell 2	2.17	489	72	0.77

Figure S1. *J-V* curves for dyes N719 and SQ2 WOPI and API.

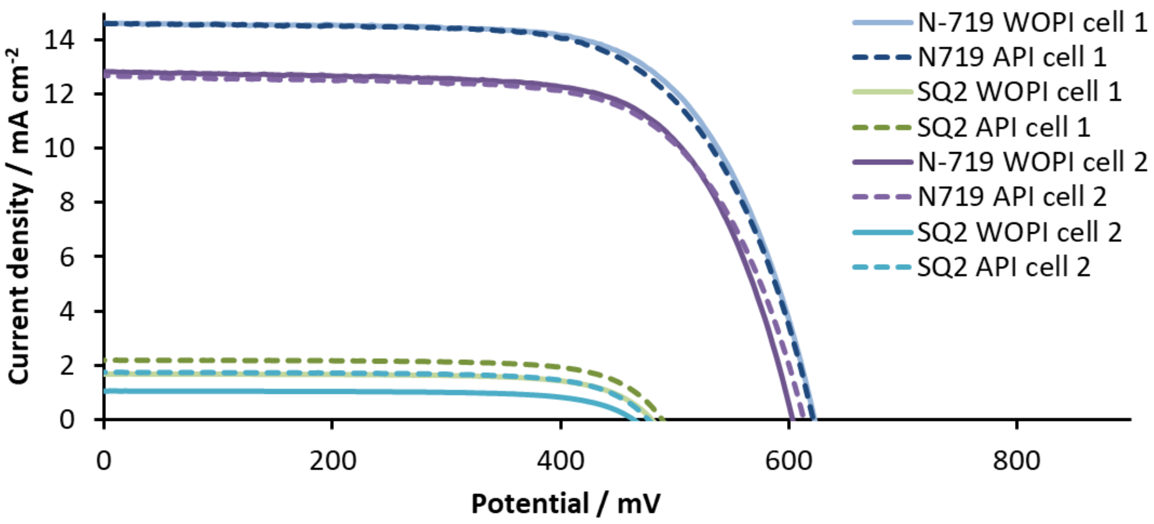

Table S2. Experimental parameters α and Q needed for the correction of CPE and the final capacitance for DSCs with N719 and SQ2 dyes WOPI and API.

DSC[1]	C_μ / μF	α	Q
N719 WOPI			1.2E-3
0 min	845	0.92	
N719 WOPI			1.3E-3
15 min	905	0.92	
N719 WOPI			1.3E-3
30 min	955	0.91	
N719 WOPI			1.4E-3
45 min	981	0.91	
N719 WOPI			1.4E-3
60 min	970	0.91	
SQ2 WOPI			3.6E-5
15 min	18	0.88	
SQ2 WOPI			4.2E-5
30 min	20	0.87	
SQ2 WOPI			4.8E-5
45 min	22	0.87	
SQ2 WOPI			5.1E-5
60 min	23	0.87	

Table S3. The EIS parameters for DSCs with N719 dye with fitting model 1.

DSC	R_{rec} / Ω	C_{μ} / μF	α	τ / ms	Ws / Ω	R_s / Ω	R_{Pt} / Ω	C_{Pt} / μF
N719 cell 1	25	759	0.93	19	12	11	11	4
N719 cell 2	22	724	0.95	16	11	11	8	4
N719 cell 3	24	902	0.94	22	9	14	7	4
N719 cell 4	22	853	0.94	19	10	11	8	5
N719 cell 5	25	850	0.93	22	11	10	8	5
N719 cell 6	24	972	0.94	24	9	11	6	5
N719 cell 7	25	957	0.95	24	14	14	7	4
N719 cell 8	23	934	0.94	21	11	10	9	4
N719 cell 9	28	955	0.94	26	11	9	7	5
N719 cell 10	27	828	0.93	22	11	14	12	4
N719 cell 11	22	756	0.94	17	10	11	8	4
N719 cell 12	17	810	0.96	14	9	12	6	4
N719 cell 13	23	670	0.95	16	10	10	7	5
N719 cell 14	25	718	0.94	18	10	10	8	4
N719 cell 15	29	615	0.95	18	9	11	7	4

Figure S2. EIS data for DSCs with N719 dye (cells 1-5).

Solid lines represent fitted curves, dotted lines represent experimental data. (a) Nyquist plots, the expansion shows the high frequency region. (b) Bode plot.

(a)

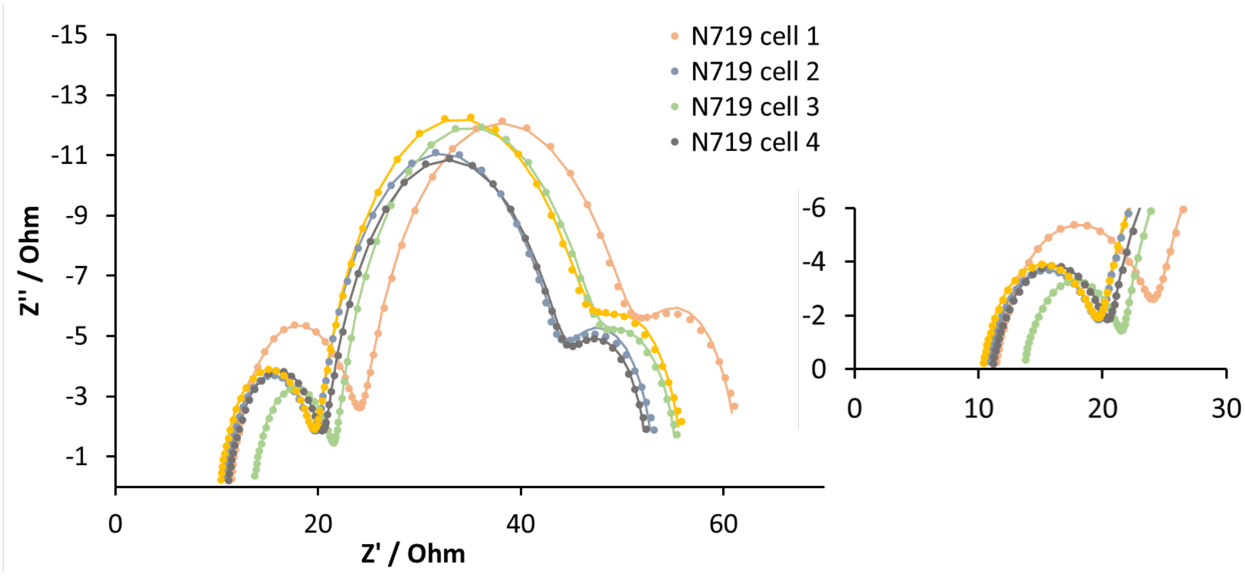

(b)

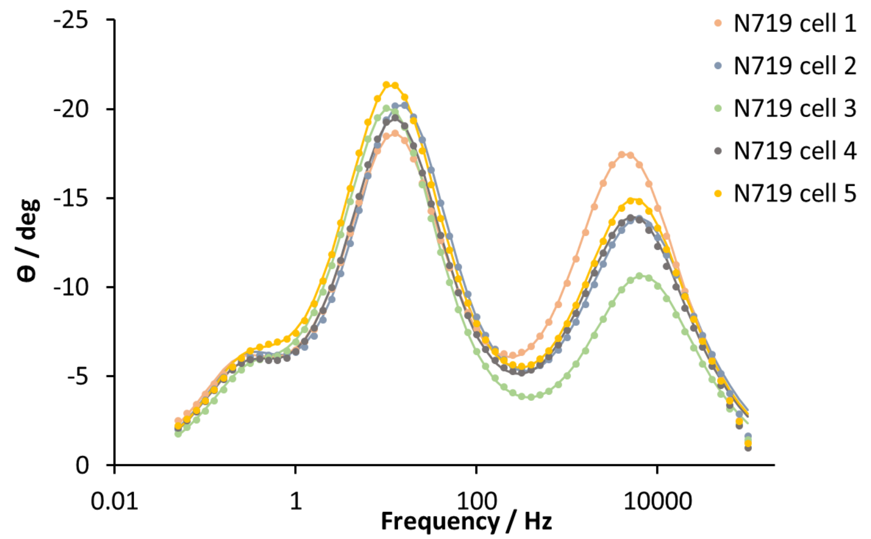

Figure S3. EIS data for DSCs with N719 dye (cells 6-10).
Solid lines represent fitted curves, dotted lines represent experimental data. (a) Nyquist plots, the expansion shows the high frequency region. (b) Bode plot.

(a)

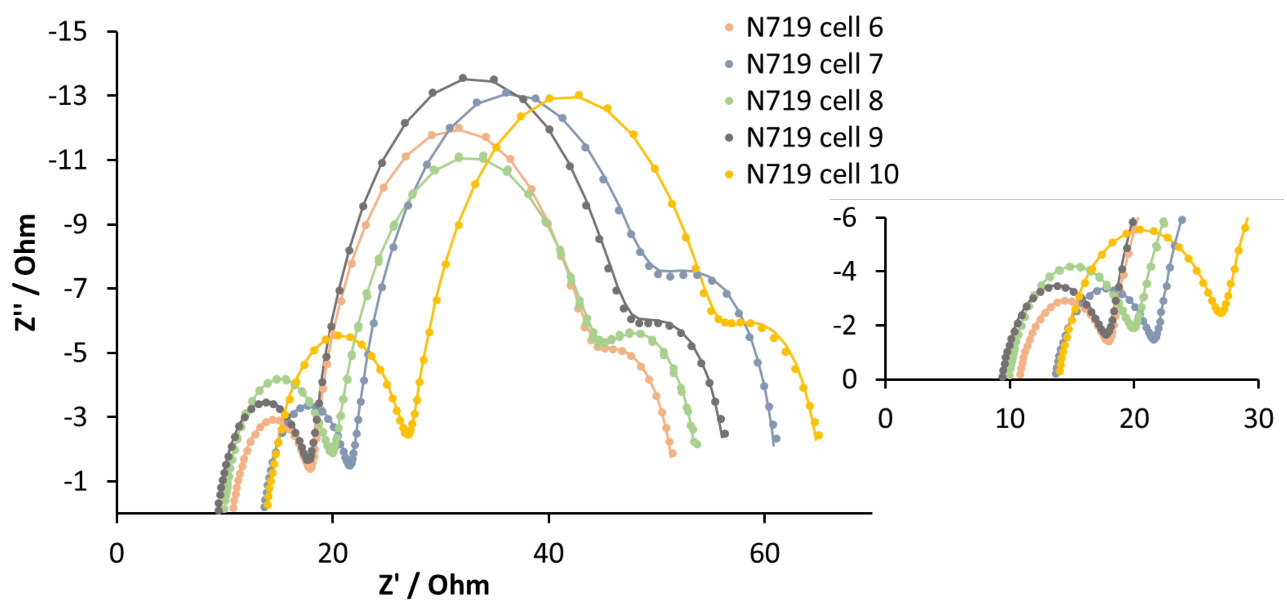

(b)

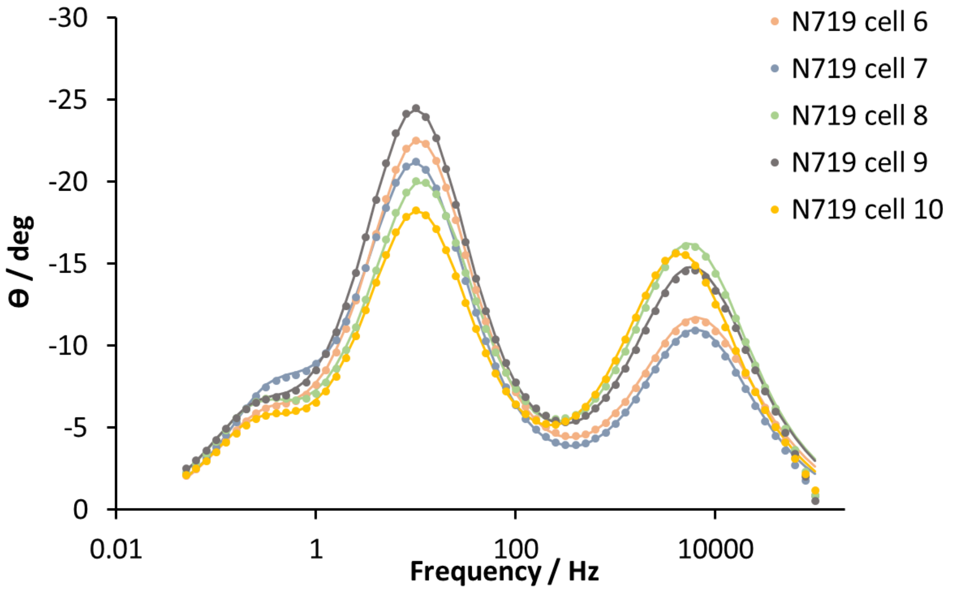

Figure S4. EIS data for DSCs with N719 dye (cells 11-15).
Solid lines represent fitted curves, dotted lines represent experimental data. (a) Nyquist plots, the expansion shows the high frequency region. (b) Bode plot.

(a)

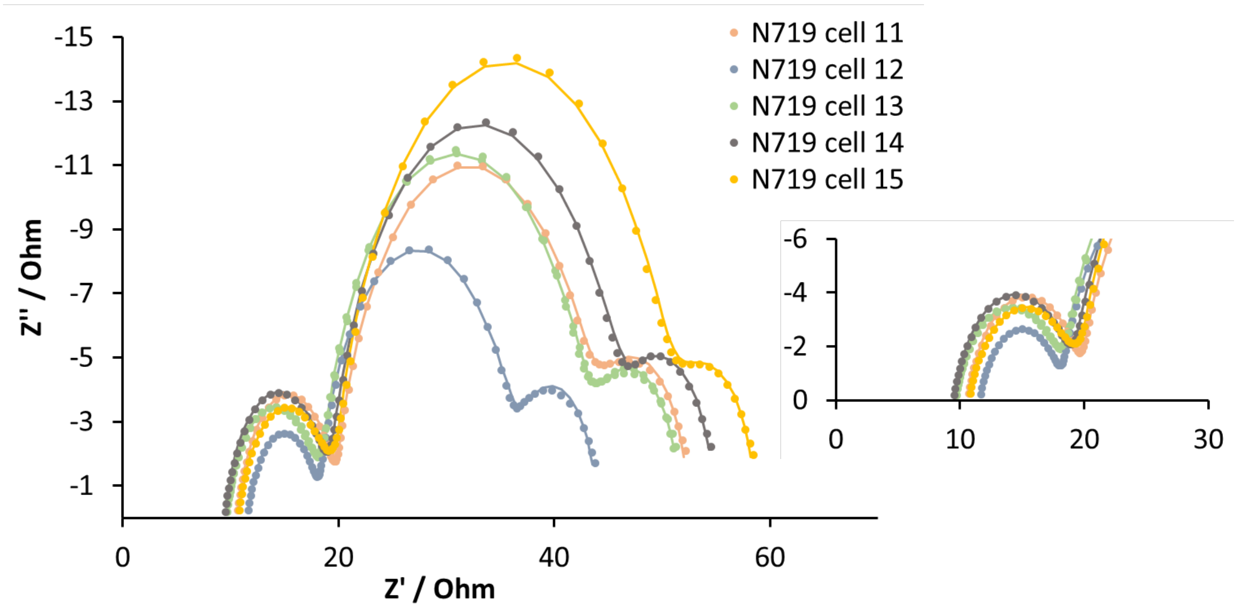

(b)

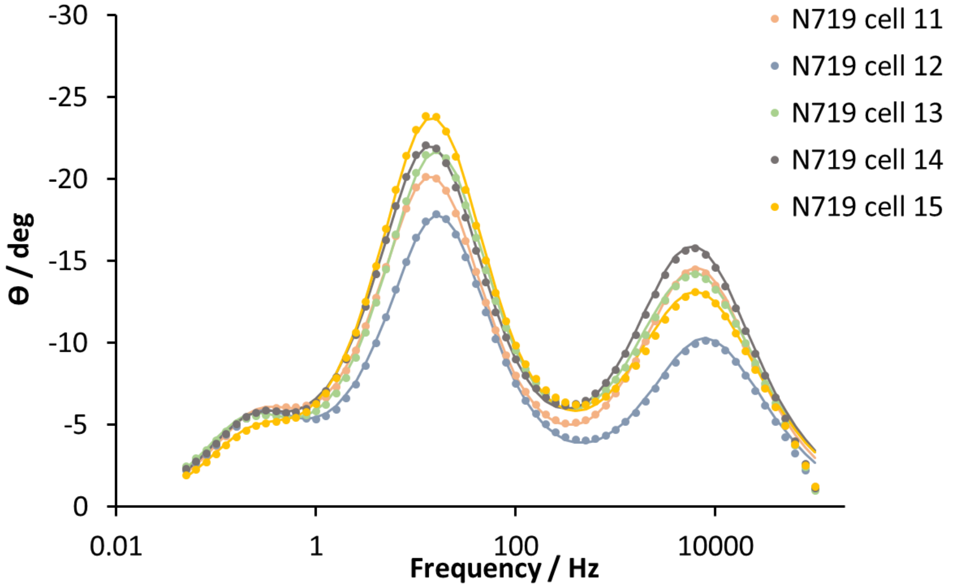

Table S4. *J-V* parameters for DSCs with N719 dye.

DSC	J_{SC} / mA cm^{-2}	V_{OC} / mV	*ff* / %	η / %
N719 cell 1	13.37	604	69	5.59
N719 cell 2	13.33	588	69	5.41
N719 cell 3	14.19	611	67	5.85
N719 cell 4	13.94	602	63	5.25
N719 cell 5	14.12	606	66	5.67
N719 cell 6	13.49	602	69	5.59
N719 cell 7	13.15	595	66	5.13
N719 cell 8	13.65	597	66	5.41
N719 cell 9	13.70	606	68	5.64
N719 cell 10	13.14	603	67	5.31
N719 cell 11	13.46	581	68	5.28
N719 cell 12	13.18	578	69	5.27
N719 cell 13	13.30	592	70	5.54
N719 cell 14	13.83	597	69	5.67
N719 cell 15	13.13	579	68	5.20

Figure S5. *J-V* curves for DSCs with N719 dye.
(a) Cells 1-5, (b) cells 6-10; (c) cells 11-15.

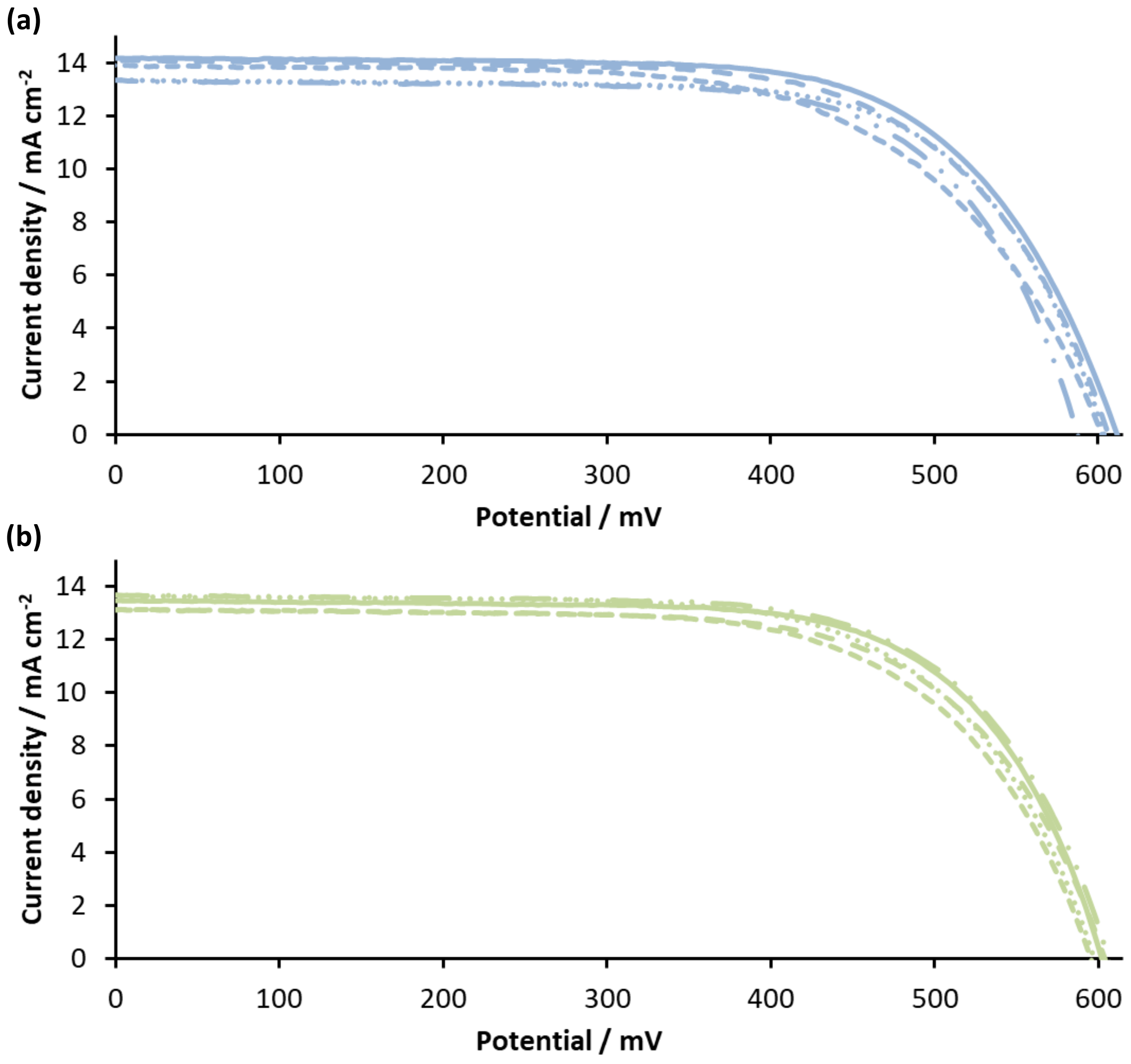

(c)

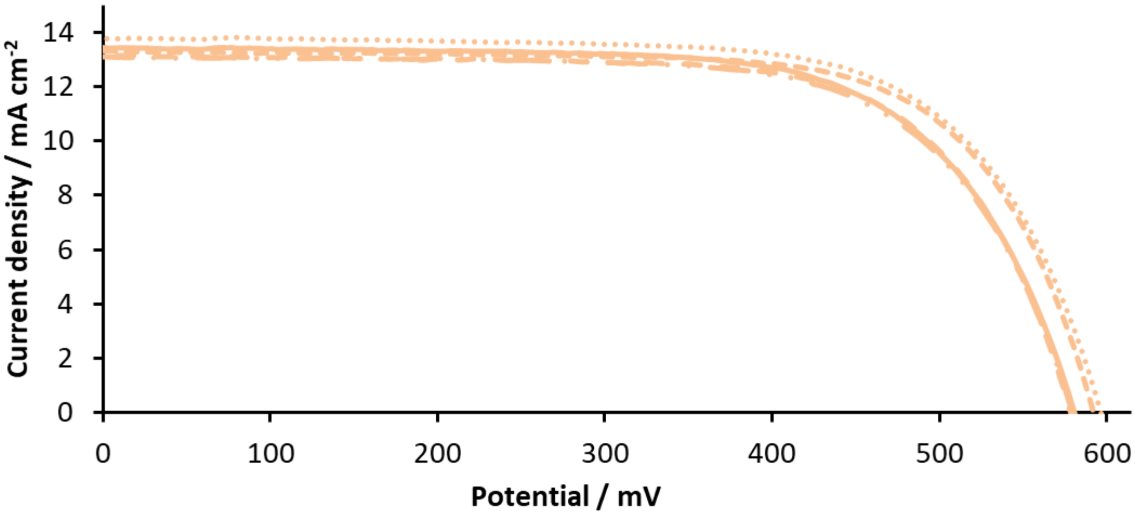

Equation S1. Average value.

$$average = \frac{x_1 + x_2 + \cdots + x_n}{n}$$

Equation S2. Standard deviation value.

$$SD = \sqrt{\frac{1}{n}\sum_{i=1}^{n}\left(n_i - n_{average}\right)^2}$$

Equation S3. Relative standard deviation value.

$$RSD = \frac{SD}{average} * 100$$

Figure S6. EIS data for DSCs with SQ2 dye (cells 1-5).
Solid lines represent fitted curves, dotted lines represent experimental data. (a) Nyquist plots, the expansion shows the high frequency region. (b) Bode plot.

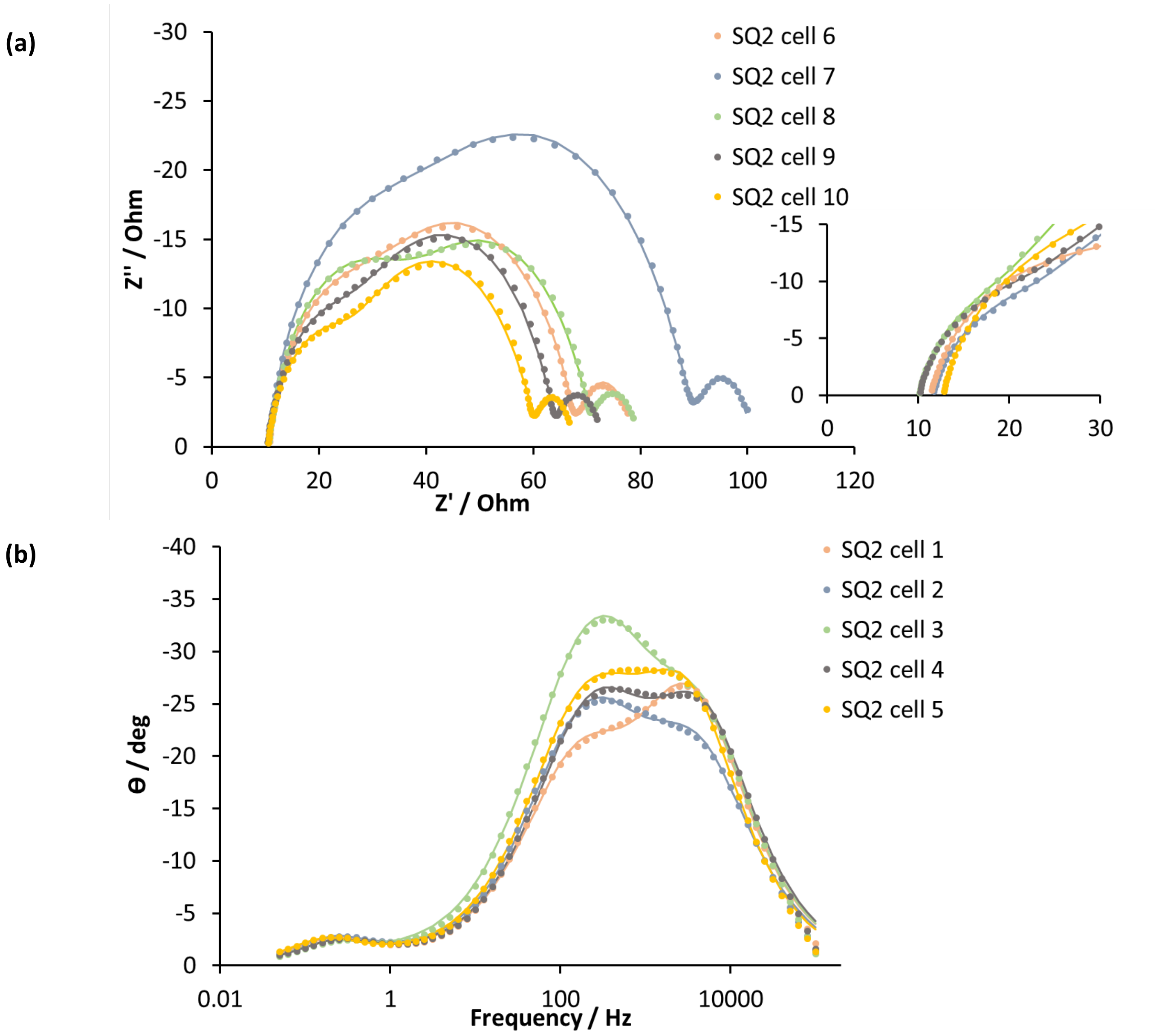

Figure S7. EIS data for DSCs with SQ2 dye (cells 6-10).
Solid lines represent fitted curves, dotted lines represent experimental data. (a) Nyquist plots, the expansion shows the high frequency region. (b) Bode plot.

(a)

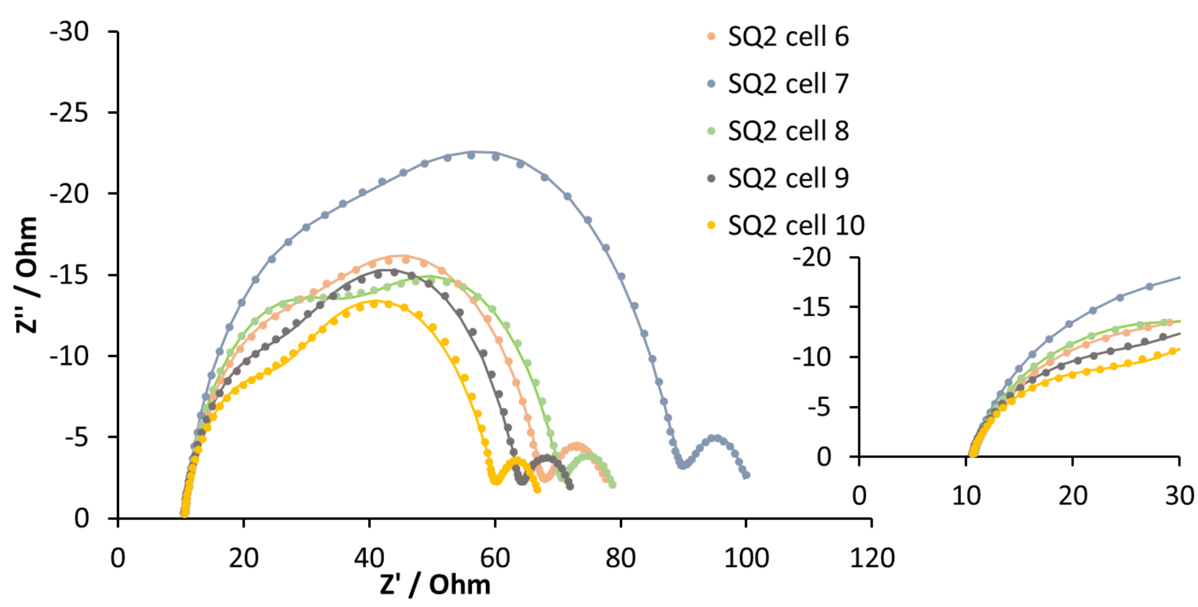

(b)

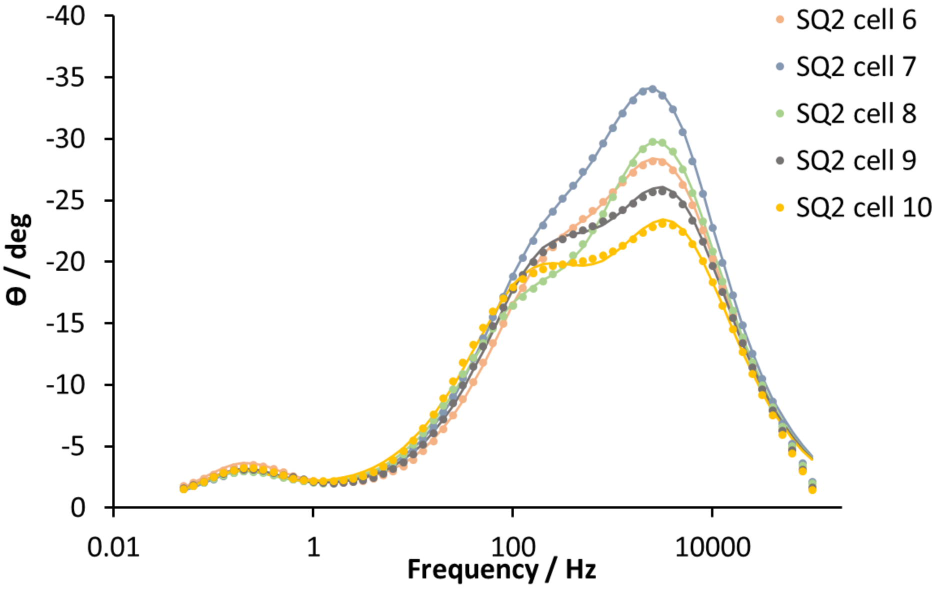

Figure S8. EIS data for DSCs with SQ2 dye (cells 11-15).
Solid lines represent fitted curves, dotted lines represent experimental data. (a) Nyquist plots, the expansion shows the high frequency region. (b) Bode plot.

(a)

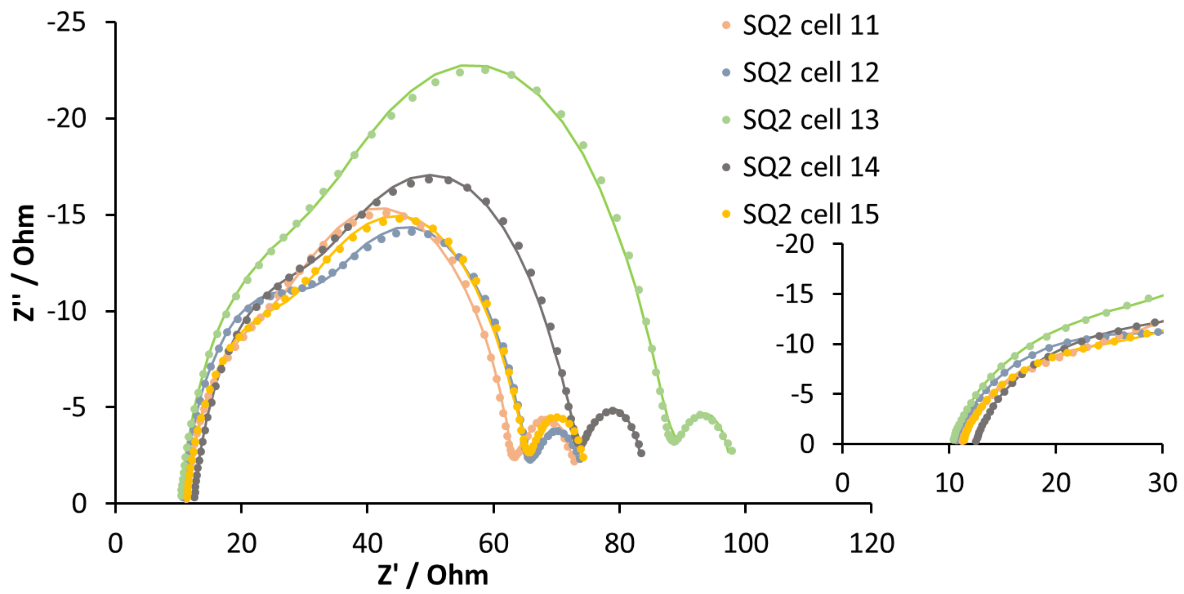

(b)

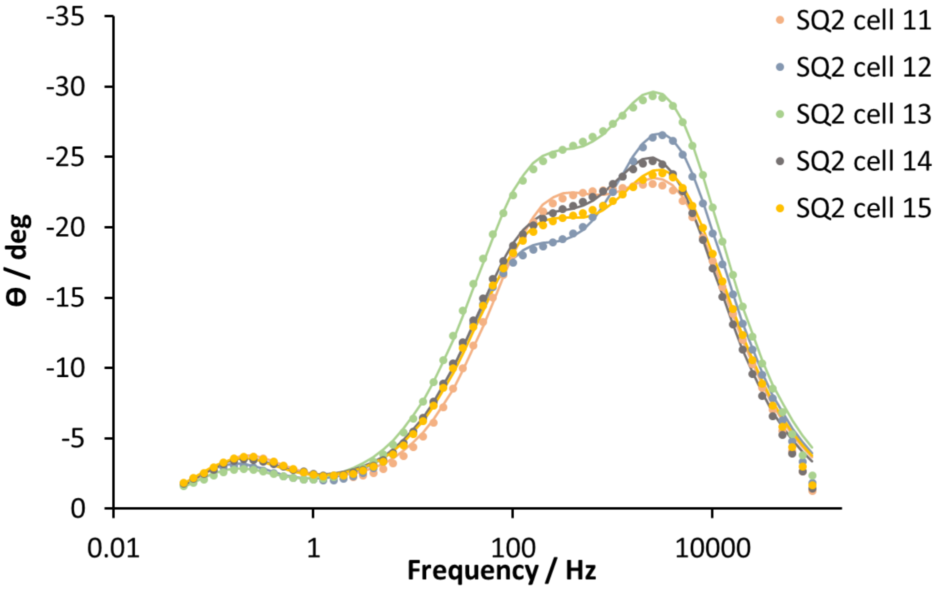

Table S5. *J-V* parameters for DSCs with SQ2 dye.

DSC	J_{SC} / mA cm^{-2}	V_{OC} / mV	*ff* / %	η / %
SQ2 cell 1	2.85	458	69	0.9
SQ2 cell 2	2.59	471	70	0.86
SQ2 cell 3	2.52	466	71	0.83
SQ2 cell 4	3.29	470	69	1.06
SQ2 cell 5	2.88	463	69	0.92
SQ2 cell 6	3.97	486	70	1.35
SQ2 cell 7	2.84	466	68	0.91
SQ2 cell 8	3.31	479	70	1.1
SQ2 cell 9	3.65	484	68	1.2
SQ2 cell 10	2.94	473	70	0.98
SQ2 cell 11	3.77	479	69	1.24
SQ2 cell 12	3.23	502	72	1.17
SQ2 cell 13	2.27	484	72	0.79
SQ2 cell 14	2.14	487	72	0.75
SQ2 cell 15	3.83	496	72	1.36

Figure S9. *J-V* curves for DSCs with SQ2 dye.

(a) Cells 1-5, (b) cells 6-10; (c) cells 11-15.

(a)

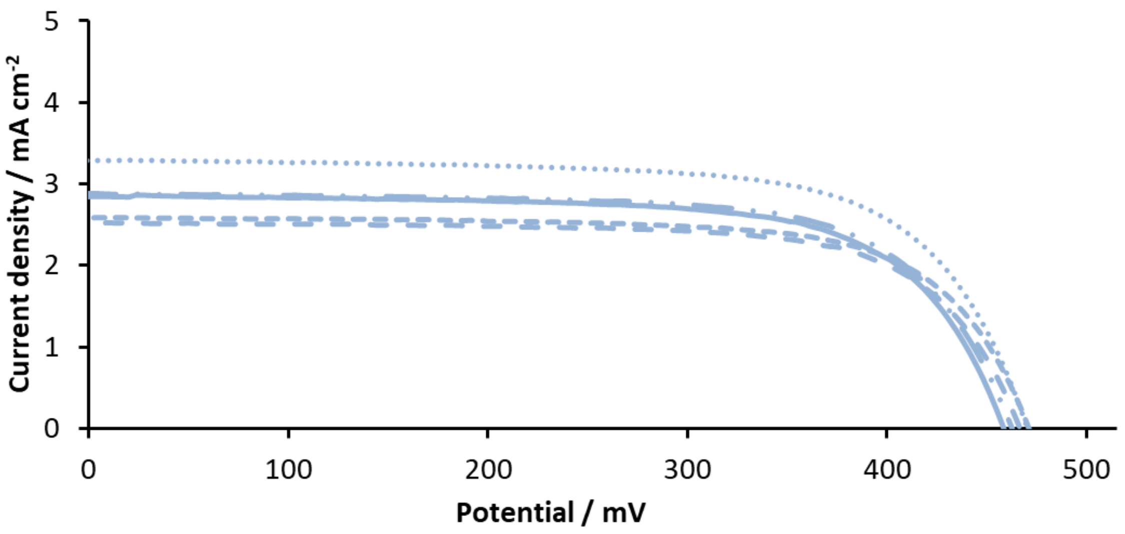

(b)

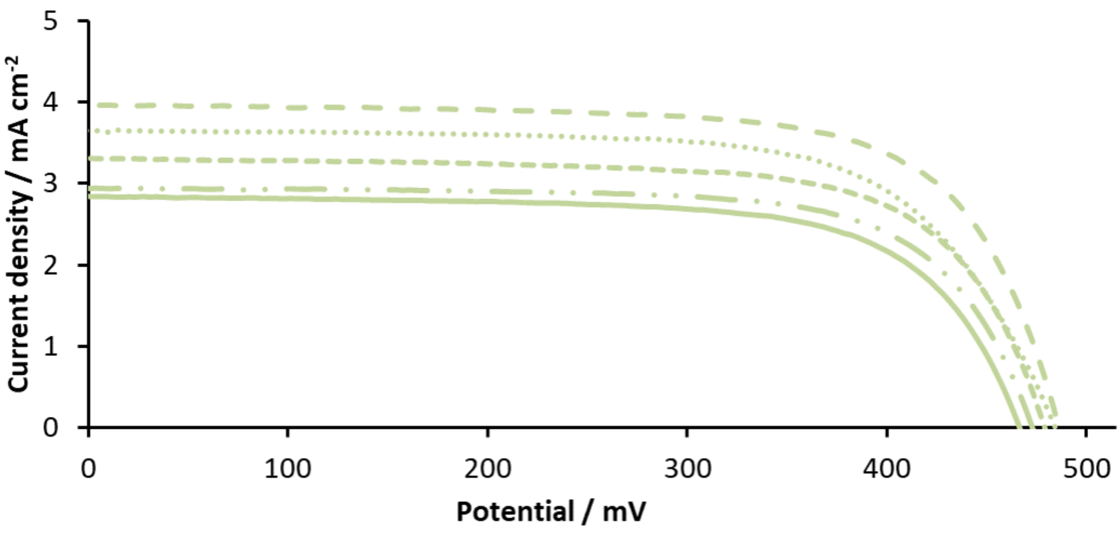
Current density / mA cm^{-2}
5
4
3
2
1
0
0
100
200
300
400
500
Potential / mV

(c)

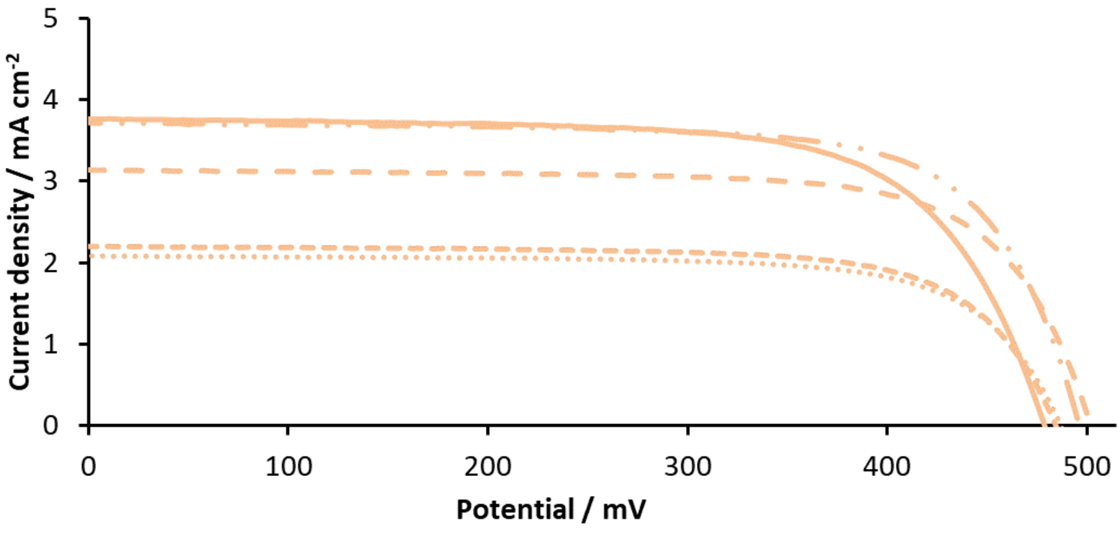
Current density / mA cm^{-2}
5
4
3
2
1
0
0
100
200
300
400
500
Potential / mV
